# Transcriptome analysis of *Macrobrachium rosenbergii* intestines under the white spot syndrome virus and poly (I:C) challenges

**DOI:** 10.1371/journal.pone.0204626

**Published:** 2018-09-28

**Authors:** Zhengfeng Ding, Min Jin, Qian Ren

**Affiliations:** 1 Institute of Aquatic Biology and Jiangsu Key Laboratory for Biofunctional Molecules, College of Life Sciences and Chemistry, Jiangsu Second Normal University, Nanjing, People’s Republic of China; 2 State Key Laboratory Breeding Base of Marine Genetic Resource, Third Institute of Oceanography, SOA, Xiamen, People’s Republic of China; 3 Co-Innovation Center for Marine Bio-Industry Technology of Jiangsu Province, Lianyungang, People’s Republic of China; 4 Jiangsu Key Laboratory for Aquatic Crustacean Diseases, College of Life Sciences, Nanjing Normal University, Nanjing, People’s Republic of China; Uppsala Universitet, SWEDEN

## Abstract

Intestine is a primary site of the white spot syndrome virus (WSSV) infection in most crustaceans. To date, little is known about its role in the anti-viral immune response in the freshwater prawn *Macrobrachium rosenbergii*. In this study, next-generation sequencing was employed to investigate the *M*. *rosenbergii* intestine transcriptomes following WSSV or poly I:C challenges. A total of 41.06 M, 39.58 M and 47.00 M clean reads were generated and assembled into 65,340, 71,241 and 70,614 transcripts from the negative control group (NG), WSSV challenge group (WG) and poly I:C treatment group (PG) respectively. Based on homology searches, functional annotation with 7 databases (NR, NT, GO, COG, KEGG, Swissprot and Interpro) for 88,412 transcripts was performed. After WSSV or poly (I:C) challenge, the numbers of up-regulated differentially expressed genes (DEGs) were greater than the down-regulated DEGs. Gene Ontology (GO) classification of the DEGs also distributed similarly, with the same top 10 annotations and were all assigned to the signaling pathways, including spliceosome, Rap1 signaling pathway, proteoglycans, PI3K-Akt signaling pathway, ECM receptor interaction. Results could contribute to a better understanding of the intestinal immune response to viral pathogens.

## Introduction

Viral diseases are thorns that affect the side of the crustacean aquaculture industry. Among those, the white spot syndrome virus (WSSV) stands out as the most devastating, causing high mortality and severe economic losses in the crustacean aquaculture industry throughout the world [1]. Almost all decapod crustaceans, including shrimps, crayfish, crab, spiny lobsters and freshwater prawns, are considered susceptible to this virus [2]. The relevance of the viral pathogen and diverse hosts still remain to be revealed. The freshwater prawn, *Macrobrachium rosenbergii*, is an economically important crustacean, being cultured on a largescale in different parts of the world. Generally, adult *M*. *rosenbergii* is considered less prone to various diseases in culture when compared to penaeid shrimps [1]. Probing into this issue may contribute towards understanding the tendency of WSSV infection and developing antiviral technologies.

*Macrobrachium rosenbergii*, like other crustaceans, possesses an innate immune system which provides defense against pathogenic agents and contains an enormous number of innate immune-related genes. We hypothesize that when *M*. *rosenbergii* infected by WSSV or treated with poly (I:C), a synthetic double-stranded RNA (dsRNA) which mimics a viral pathogen-associated molecular patterns (PAMP), these genes should be synergistically mobilized to play their respective roles in defense, especially in the humoral immune response [3]. Elucidation of the specificity will be helpful for understanding the WSSV infection mechanisms. Recently, there have been several reports of the transcriptome sequencing of *M*. *rosenbergii* tissues such as muscle, hepatopancreas, ovary, testis, spermary, lymphoid organ, gill and stomach [3–5]. Intestine, as a complex ecosystem containing a diverse pathogenic community, plays an important role in removing invading pathogens via an efficient and specific immune pattern. More importantly, ingestion of WSSV-infected prawn has been accepted as the major route of natural infection due to the cannibalistic nature of many crustaceans [6]. To our knowledge, no studies have been reported on the intestine transcriptome of *M*. *rosenbergii* in response to WSSV or the viral PAMP mimic (poly I:C) challenge.

Therefore, *de novo* transcriptome sequencing of the prawn intestine following WSSV or poly (I:C) challenge was carried out, and a global survey of immune-related genes, annotation of immune signaling pathways and determination of gene expression were also performed. Furthermore, putative simple sequence repeats (SSRs) and single nucleotide polymorphisms (SNPs) were analyzed. These results provided the first experimental access to *M*. *rosenbergii* intestine-specific genes involved in the anti-viral intestine immune response and could serve as the basis for additional in-depth molecular and genomic analyses.

## Materials and methods

### Preparation of *M*. *rosenbergii* intestines and immune challenge

*M*. *rosenbergii* (body weight 9–12 g) were purchased from a commercial aquaculture market in Nanjing, Jiangsu Province, China. The prawns were acclimatized for 1 week in tanks (300 L) with aerated and filtered freshwater at 27 ± 1 °C in the laboratory. They were then randomly sampled and tested by polymerase chain reaction (PCR) to ensure they were free from WSSV [7]. Three groups were then divided: WSSV challenge group (WG), poly (I:C) challenge group (PG) and negative control group (NG). Each group contained 30 prawns.

In order to ensure each prawn was infected successfully and control the virus concentration more accurately, the injection model was used in the challenge experiments. WSSV was propagated by inoculation of clarified gill homogenates from previously infected *M*. *rosenbergii*. The gill tissues (6 g) were homogenized in 10 ml PBS (pH 7.4) and clarified by centrifugation at 10,000 *g* for 25 min at 4 °C. The supernatant was then filtered through a 0.22 μm filter. WSSV viral load was quantified using the real-time PCR technique [8]. The WSSV solution, serially diluted to 100 copies μl^-1^ with PBS, was used as inocula. Each prawn was intramuscularly injected with 100 μl WSSV solution in WG. The PG prawns were injected of 5 μg poly (I:C) per 1 g body weight, while the NG prawns were injected with 100 μl of PBS (pH 7.4) [9].

Then, 48 h after challenge, hindgut of the intestine was collected from 10 prawns of each group, frozen immediately in liquid nitrogen for total RNA extraction and preserved in 75% alcohol for WSSV PCR detection to confirm viral infection after the challenge. In this study, the hindgut was dissected out for analysis as that was the easiest one to obtain in dissection of prawns.

### RNA isolation and sequencing

Total RNA was extracted from WG, PG and NG samples using a high-purity total RNA Rapid Extraction Kit according to the manufacturer’s instructions. Total RNA quality was checked on 1% formaldehyde agarose gel via electrophoresis, and RNA concentration was determined through Nano Drop. Then approximately 5 μg of total RNA after the on-column DNase treatment was used to construct a cDNA library following the protocols of the RNA Sample Preparation Kit. After necessary quantification and qualification, the library was sequenced with 100 bp paired-end reads for WG, PG and NG respectively.

### *De novo* assembly and data analysis

The raw reads were processed by Sickle (https://github.com/najoshi/sickle) and SeqPrep (http://github.com/jstjohn/SeqPrep) with default parameters and sequences under 60 bases were eliminated. RNA assembly of clean reads was done by using Trinity program [10]. By BLAST algorithms, the assembled contigs were furtherly annotated. The unigenes were aligned by a BLASTx search, the function annotations of which were retrieved based on the highest sequence similarity and using an *E*-value cut-off of 10^−5^ [11]. The best alignment results were used to determine the sequence direction and protein-coding-region prediction. The Blast2GO suite [12] and KEGG database [13] were applied to obtain GO annotations and the complex biological behavior of the uniquely assembled transcripts.

Microsatellite search module (MISA http://pgrc.ipk-gatersleben.de/misa/) was used to find simple sequence repeats (SSRs) in unigenes, then design primer for each SSR [14]. All clean reads were mapped to unigenes using HISAT (hierarchical indexing for spliced alignment of transcripts), then call single nucleotide polymorphisms (SNPs) with Genome Analysis Toolkit (GATK). After filter out the unreliable sites, the final SNP was gotten in VCF format.

### Analysis of DEGs (differentially expressed genes)

To estimate the expression level of each transcript, fragments per kilobase of transcripts per million fragments mapped (FRKM) was applied as the unit of measurement. FDR (False discovery rate) was used as corrections of *E*-value. Genes with FDR≤0.001 and an FPKM ratio larger than 2 or smaller than 0.5 were considered as differentially expressed genes (DEGs) between samples. NG vs WG and NG vs PG were compared respectively. With DEGs, we performed Gene Ontology (GO), KEGG pathway classification and functional enrichment.

## Results

### Sequencing and *de novo* assembly

A total of 41.05 M clean reads that represent 6.16 Gb clean bases were generated for NG (negative control group). For WG (WSSV-infected group), 39.58 M clean reads that represent 5.94 Gb clean bases were generated. While for PG (poly I:C treatment group), a total of 47.00 M clean reads were obtained, thereby providing a total of 7.05 Gb clean bases. The GC content of nucleotide was 39.56%, 39.43% and 39.49% respectively. Transcriptome assembly created 65,340, 71,241 and 70,614 transcripts with a mean length of 973, 937 and 1016 nucleotides for each group (Table 1).

**Table 1:** Summary of sequencing reads after filtering and quality metrics of unigenes. NG (negative control group), WG (WSSV-infected group) and PG (poly I:C challenge group).

Sample	Total Clean Reads (Mb)	Total Clean Bases (Gb)	GC (%)	Total Unigenes Number	Mean Length	N50
NG	41.05	6.16	39.56	65340	973	2185
WG	39.58	5.94	39.43	71241	937	2099
PG	47	7.05	39.49	70614	1016	2355

### Functional annotation and classification of transcriptome sequences

To achieve protein identification and gene annotation, a search was made on standard unigenes in the NCBI non-redundant (Nr) (26,432 unigenes, 29.90%), Nt (17,091 unigenes, 19.33%), Swiss-Prot (21,491 unigenes, 24.31%), KEGG (21,446 unigenes, 24.26%), clusters of orthologous groups of proteins (COGs) (11,650 unigenes, 13.18%), Interpro (19,653 unigenes, 22.23%) and GO (4,544 unigenes, 5.14%) using the BLAST program (*E*-value <10^−5^). This search yielded a total of 32,717 significant hits (37.01% of all unigenes).

**Fig 1:**
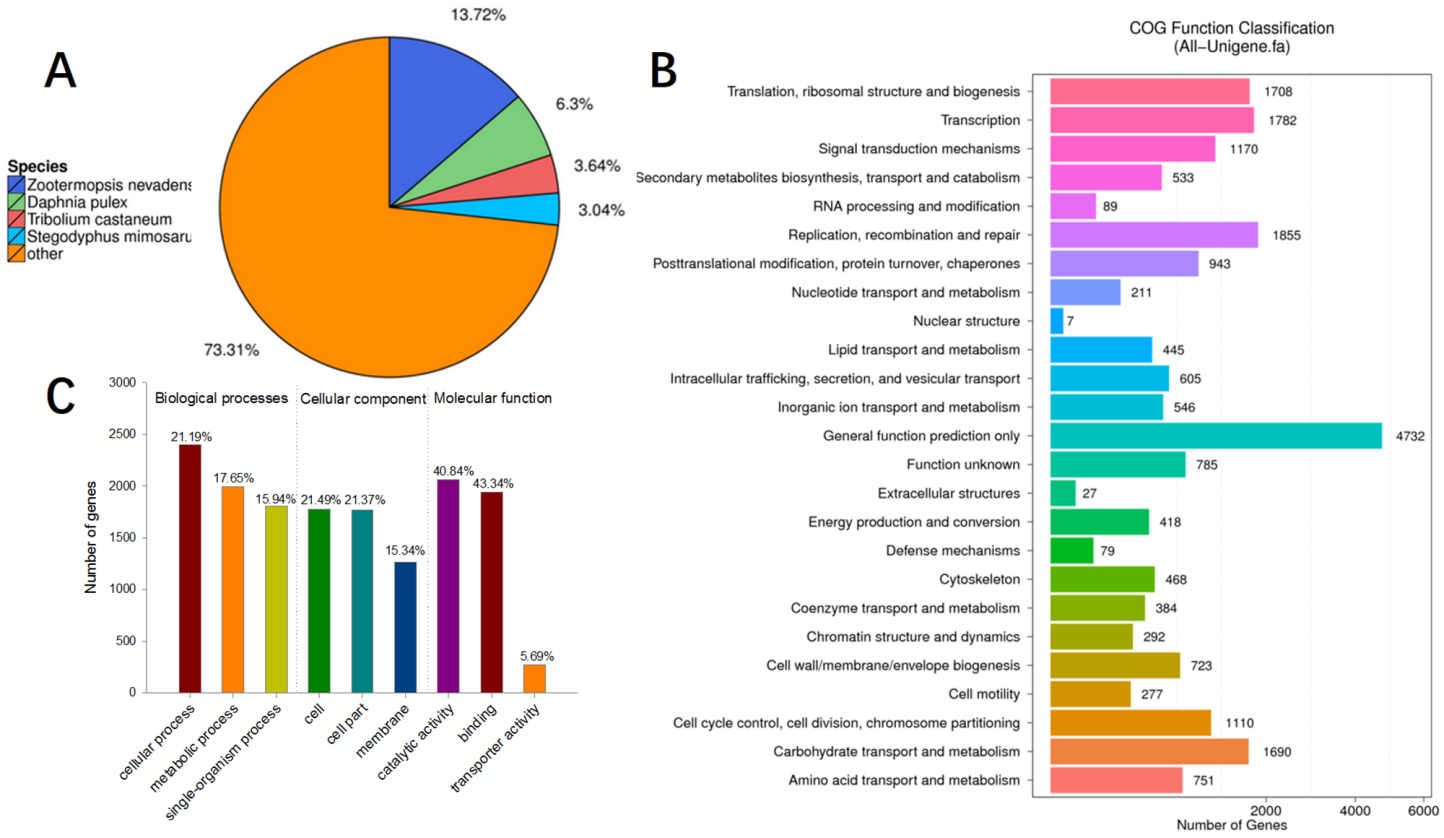
Functional annotation and classification of *Macrobrachium rosenbergii* intestine transcriptome sequences. (A) Species distribution of the BLASTx matches of the transcriptome unigenes. This figure showed the species distribution of unigene BLASTx matches against the Nr protein database (cut-off value *E* < 10–5) and the proportions for each species. (B) Functional distribution of COG annotation. (C) Top 10 of the GO annotations.

Fig 1A showed the species distribution of unigene BLASTx matches against the Nr protein database (cut-off value *E* < 10^−5^) and the proportions for each species. About 26.7% of the total unigenes matched with sequences from four top-hit species, i.e., *Zootermopsis nevadensis*, *Daphnia pulex*, *Tribolium castaneum* and *Stegodyphus mimosarum*, all of which belonged to arthropoda.

The standard unigenes were then aligned to the COG database to predict their potential roles. A total of 11,650 unigenes distributed among 25 COG categories, including “replication", “recombination and repair”, “signal transduction mechanisms”, “cell wall/membrane/envelope biogenesis”, “post-translation modification, protein turnover, chaperones”, all of which play important roles in virus pathogenesis (Fig 1B).

Sequence homology based on GO classification revealed that 4,544 annotated unigenes were assigned to three GO categories, including 54 functional groups. A total of 24,394 GO assignments, where 46.38% comprised biological processes, 33.92% comprised cellular component, and 19.51% comprised molecular function. (Fig 1C).

KEGG analysis indicated that a greater number of genes expressed in the prawn intestine were associated with human disease (26.59%), metabolism (19.24%) and organismal systems (20.99%). The largest one was “signal transduction” (3571, 9.34%). The second largest was “global and overview maps” (2,770, 7.24%); followed by “cancers: overview” (2,299, 6.01%); “endocrine system” (1,590, 4.16%); and “transport and catabolism” (1,510, 3.95%). The two smallest groups were “Biosynthesis of other secondary metabolites” (25, 0.65%) and “Metabolism of terpenoids and polyketides” (65, 0.17%) (S1 Fig).

### SSRs/SNP markers identification

**Fig 2:**
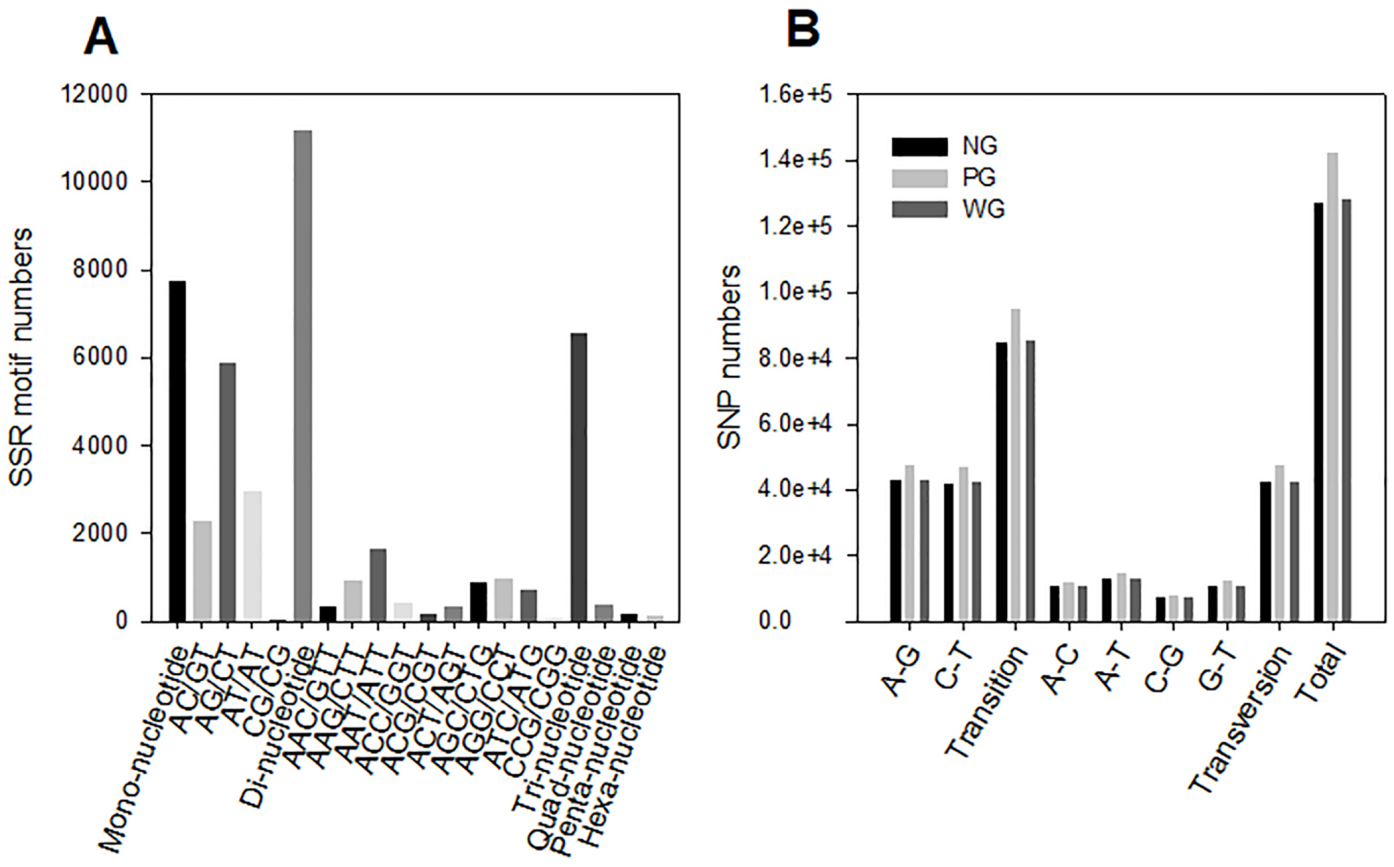
A summary of the simple sequence repeats (SSRs) and single nucleotide polymorphisms (SNP) markers identified from the *Macrobrachium rosenbergii* intestinal transcriptome. (A) Distribution of SSRs based on different motif types; (B) Distribution of SNP 48 h after WSSV and Poly I:C challenges (SNP variant type distribution).

A total amount of 52,316 SSRs were identified from the *M*. *rosenbergii* intestine gene library. Dinucleotide, mononucleotide, and trinucleotide repeats were the three largest SSRs, accounting for 42.73%, 29.63%, and 21.19% of all SSRs, respectively. The dominant repeat motif was AG/CT (5867, 11.21%), followed by AT/TA (2981, 5.69%), and AC/GT (2295, 4.38%) in all the SSRs (Fig 2A).

The three transcriptomes of WG, PG and NG were similar within the detected SNPs. Transitions were much more common than transversions. Similar percentages of four transversion types (A/T, A/C, G/T, C/G) and numbers of C/T and A/G transitions were detected (Fig 2B).

### Identification of differentially expressed genes

**Fig 3:**
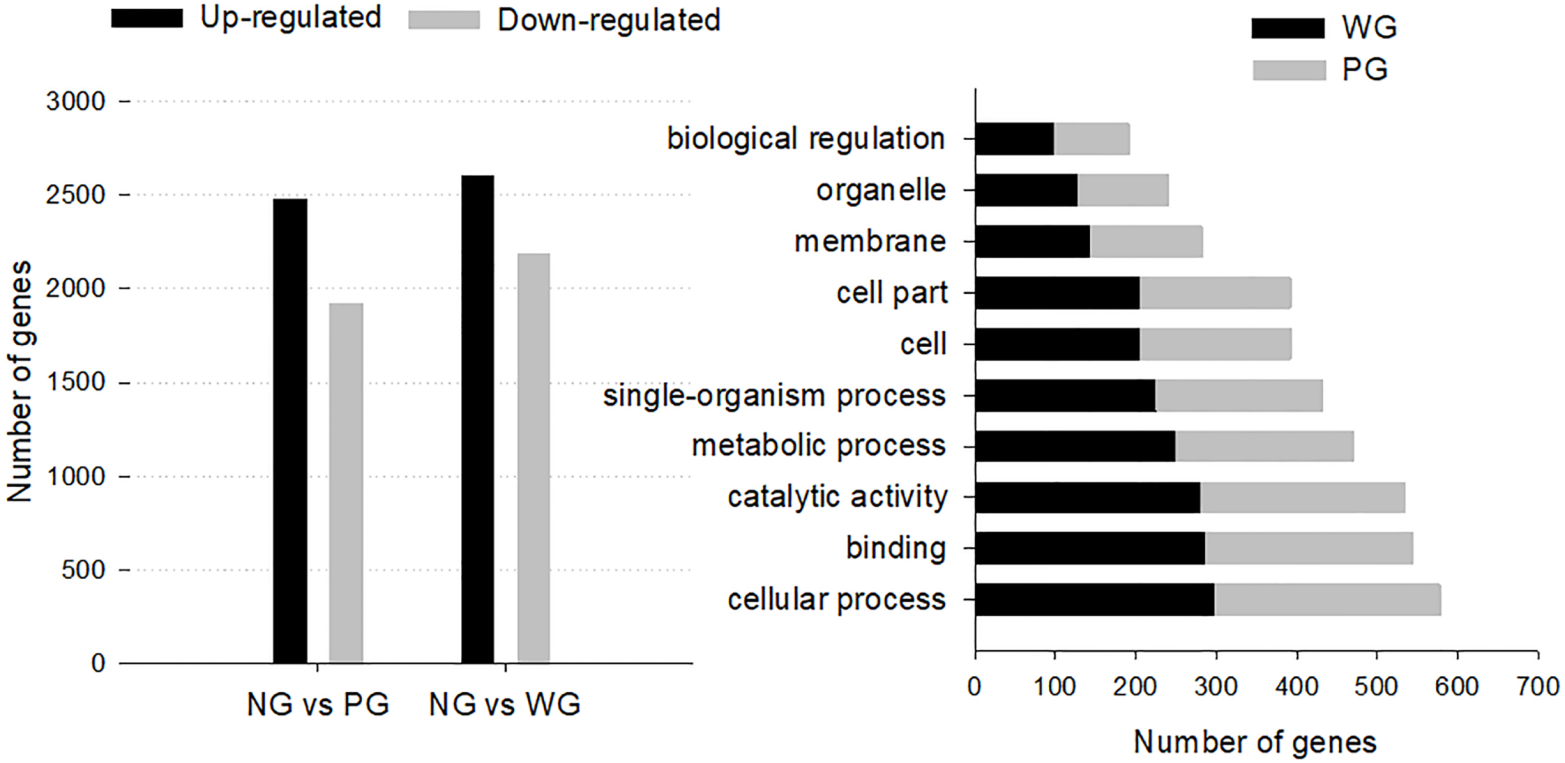
A, static of differentially expressed genes (DEGs) 48 h post infections of WSSV and poly I: C. B, functional distribution and Top 10 of the GO classification of DEGs.

Previous sequence analysis and annotation for all of the unigenes in the merged group (NG, WG and PG) provided some valuable information to analyze the prawn intestine transcriptome. However, the variation in the gene expression level after WSSV or poly I:C challenge was expected. Following WSSV infection, 2604 genes were up-regulated and 2192 genes down-regulated. In comparison, after poly (I:C) treatment, 2480 genes were up-regulated and 1928 genes down-regulated (Fig 3A). The up-regulated DEGs were all much greater than down-regulated DEGs, thereby indicating that the WSSV and poly (I:C) challenge processes were associated with transcript accumulation. The DEG distributions after WSSV or Poly I:C treatment were similar, as showed in the MA plots (Figs A and B in S2 File) and hierarchical clustering (Fig C in S2 File).

DEGs that were annotated in the GO database were categorized into 52 functional groups and distributed similarly in response to WSSV or poly (I:C) challenge, with the same top 10 annotations: “binding”, “catalytic activity”, “biological regulation”, “cellular process”, “metabolic process”, “single-organism process”, “cell”, “cell part”, “membrane” and “organelle” (Fig 3B), most of which were covered by the combined annotation as shown in Fig 1C.

**Fig 4:**
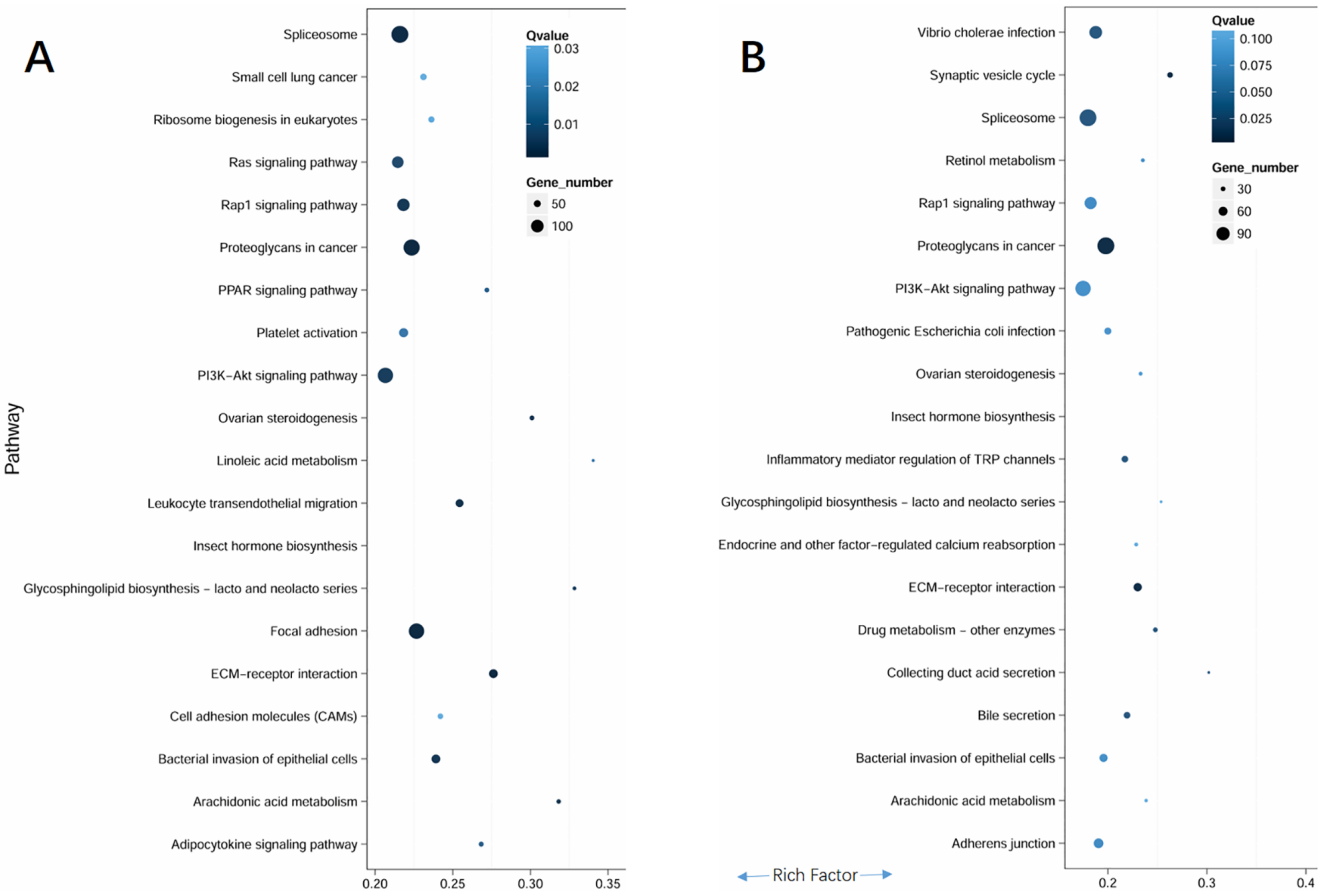
Scatter diagram of pathway enrichment for differentially expressed genes (DEGs) following 48 h infection of WSSV (A) and poly I:C (B). The top 20 pathways were listed.

Significantly, following WSSV or poly (I:C) challenge, DEGs were consistently assigned to comprehensive host defense signaling pathways, which were related to various antiviral responses, such as “spliceosome”, “Rap1 signaling pathway”, “proteoglycans”, “PI3K-Akt signaling pathway”, “ECM receptor interaction” (Fig 4) A detailed explanation was presented in the discussion and selected immune involved genes were listed in Table 2. In addition, after WSSV challenge, DEGs were also related to “Ras signaling pathway”, “platelet activation”, “leukocyte transendothelial migration”, “focal adhesion”, “cell adhesion molecules (CAMs)”, “bacterial invasion of epithelial cells”. In comparison, following poly (I:C) treatment, DEGs were also involved in different pathways of “adherence junction”; “bacterial invasion of epithelial cells”; “inflammatory mediator regulation of TRP channels”; “*Vibrio cholera* infection” (Fig 4). The different responsive pathways indicated that WSSV and the synthetic viral analogue poly (I:C) could induce some different host immune reactions.

**Table 2:** Selected intestine-specific DEGs potentially involved in *Macrobrachium rosenbergii* immune response against viral challenge.

Category or gene id	Homologues function	Species	FC^a^
*Spliceosome*			
Unigene3652_All	snRNP core protein D3-like protein	*Scylla paramamosain*	11.1
Unigene13614_All	small nuclear ribonucleoprotein polypeptide G	*Cherax quadricarinatus*	8.3
CL4598.Contig2_All	U1 small nuclear ribonucleoprotein	*Diaphorina citri*	-7.6
CL4134.Contig1_All	ATP-dependent RNA helicase	*Macrobrachium nipponense*	1.8
CL2220.Contig1_All	Asp-Glu-Ala-Asp) box polypeptide	*Macrobrachium nipponense*	1.5
*Rap1 signaling pathway*			
CL186.Contig4_All	GTP binding protein alpha subunit Gi	*Marsupenaeus japonicus*	-6.2
Unigene67212_All	Troponin C, isoform 1	*Homarus americanus*	-7.5
Unigene23813_All	Crustacean calcium-binding protein 23	*Orconectes limosus*	-1.9
Unigene876_All	phospholipid phospholipase C	*Homarus americanus*	-3.6
CL1728.Contig2_All	atrophin-1 interacting protein 3	*Zootermopsis nevadensis*	-7.5
CL3363.Contig2_All	Techylectin-5B	*Zootermopsis nevadensis*	-7.1
CL4616.Contig1_All	ficolin-like protein 2	*Pacifastacus leniusculus*	-2.7
Unigene31718_All	RAP1 GTPase activating protein 1	*Tribolium castaneum*	9.1
CL493.Contig1_All	Ras-associated and pleckstrin-like proteiny domains-containing protein 1	*Zootermopsis nevadensis*	-9.1
Unigene56967_All	skeletal muscle actin 6	*Homarus americanus*	-4.2
Unigene13026_All	actin 1	*Penaeus monodon*	-1.9
CL1651.Contig1_All	tyrosine-protein kinase Src	*Zootermopsis nevadensis*	6.5
CL2362.Contig1_All	partitioning defective protein 3	*Tribolium castaneum*	7.3
CL1190.Contig11_All	cadherin-associated protein	*Zootermopsis nevadensis*	10.0
CL1977.Contig4_All	p38 MAP kinase	*Litopenaeus vannamei*	1.5
CL5481.Contig3_All	GTPase HRas	*Marsupenaeus japonicus*	2.2
*Proteoglycans*			
CL607.Contig15_All	cortactin	*Crassostrea gigas*	-1.1
CL5920.Contig3_All	threonine-protein kinase PAK 3	*Zootermopsis nevadensis*	8.1
CL221.Contig21_All	Ankyrin-3, partial	*Stegodyphus mimosarum*	9.4
CL1339.Contig1_All	ANK-like protein	*Eriocheir sinensis*	-7.0
Unigene17362_All	inositol 1,4,5-trisphosphate receptor	*Panulirus argus*	7.0
CL1538.Contig2_All	CaM kinase II	*Periplaneta americana*	3.0
CL138.Contig4_All	ATP-dependent RNA helicase	*Macrobrachium nipponense*	4.1
CL5421.Contig2_All	ribonuclease III	*Marsupenaeus japonicus*	1.6
CL3885.Contig3_All	integrin beta 1	*Litopenaeus vannamei*	6.4
Unigene5486_All	integrin alpha V	*Daphnia pulex*	-3.3
CL5448.Contig2_All	radixin	*Daphnia pulex*	-1.8
CL1983.Contig10_All	focal adhesion kinase	*Marsupenaeus japonicus*	7.4
CL1139.Contig20_All	heparan sulfate proteoglycan 2	*Tribolium castaneum*	6.7
CL1977.Contig4_All	p38 mitogen-activated protein kinase	*Litopenaeus vannamei*	1.5
“PI3K-Akt signaling pathway”			
CL5874.Contig1_All	protein phosphatase 2A regulatory subunit B	*Scylla paramamosain*	4.0
Unigene25378_All	pacifastin-related serine protease inhibitor	*Portunus trituberculatus*	8.8
Unigene3862_All	hemolectin	*Macrobrachium rosenbergii*	-2.6
Unigene23817_All	fibrinogen-related protein 1	*Marsupenaeus japonicus*	1.6
CL4616.Contig1_All	ficolin-like protein 2	*Pacifastacus leniusculus*	-2.7
Unigene1011_All	Protein charybde	*Zootermopsis nevadensis*	-2.2
Unigene12442_All	translation initiation factor 4E	*Amblyomma variegatum*	3.8
Unigene16482_All	angiopoietin 1	*Branchiostoma floridae*	3.5
CL4483.Contig4_All	guanine nucleotide-binding protein subunit beta-4	*Ixodes scapularis*	3.3
CL4726.Contig3_All	phosphoenolpyruvate carboxykinase	*Litopenaeus vannamei*	-8.8
CL2352.Contig1_All	FoxO protein	*Blattella germanica*	-6.6
CL3508.Contig8_All	putative MhmaT1 transposase	*Misgolas hubbardi*	8.9
CL4686.Contig2_All	IKKbeta	*Litopenaeus vannamei*	2.9
CL1113.Contig2_All	nuclear factor NF-kappa-B p105	*Litopenaeus vannamei*	-1.5
“ECM receptor interaction”			
Unigene27154_All	Kazal-type serine proteinase inhibitor	*Fenneropenaeus chinensis*	-5.4
CL1139.Contig20_All	heparan sulfate proteoglycan 2	*Tribolium castaneum*	6.7
CL2931.Contig2_All	hemocytin	*Tribolium castaneum*	-7.0
Unigene58062_All	dystroglycan 1	*Daphnia pulex*	-7.9
Unigene7662_All	hemolectin	*Papilio xuthus*	-2.4
CL2493.Contig1_All	Leukocyte receptor cluster member 9	*Caligus rogercresseyi*	-1.4
Unigene28540_All	protein kinase C	*Cerapachys biroi*	-1.4
CL4616.Contig1_All	ficolin-like protein 2	*Pacifastacus leniusculus*	-2.7
Focal adhesion			
Unigene59920_All	myosin light chain 2	*Procambarus clarkii*	-5.9
CL1581.Contig1_All	p21-activated kinase 7	*Nasonia vitripennis*	1.9
CL5920.Contig3_All	threonine-protein kinase PAK 3	*Zootermopsis nevadensis*	8.1
Metabolic pathways			
CL3548.Contig5_All	phosphofructokinase	*Litopenaeus vannamei*	9.66
Unigene16161_All	mucin-19-like isoform X7	*Apis mellifera*	3.12
MAPK signaling pathway			
CL3045.Contig3_All	protein phosphatase 1B	*Marsupenaeus japonicus*	9.2

^a^ Fold changes (log2 ratio) in gene expression.

All the raw data including the expressed gene lists and the differentially expressed genes (DEGs) lists were supplemented in the Dryad Digital Repository: https://doi.org/10.5061/dryad.53f1j4d.

## Discussion

The innate immune response of invertebrate intestine is a crucial defense mechanism against external pathogens [15]. For *M*. *rosenbergii*, intestine is also the primary site of WSSV infection [6] and a likely site of differential gene expression following infection. In order to clearly elucidate its antiviral mechanism, we analyzed the transcriptomes of *M*. *rosenbergii* intestine after WSSV and viral PAMP (poly I:C) treatments using high throughput sequencing technology (RNA-seq), which could provide enormous amounts of sequence data in a much shorter amount of time and at a much cheaper cost. To date, transcriptome data for *M*. *rosenbergii* intestine in response to WSSV or poly I:C challenge has not been reported.

Poly I:C has been widely applied in mimicking viral infection and elucidating host immune response and gene expression [9]. Our results confirmed that poly (I:C) stimulated a defense state in *M*. *rosenbergii* and could be a powerful inducer of putative antiviral gene expression in the prawn. DEGs of WSSV and poly I:C treatments distributed in a similar manner, but still presented some unique characteristics including the different numbers of up or down-regulated genes and different GO classification and pathways enrichment. These results were consistent with some previous studies, which demonstrated that several important immune genes (Myeloid differentiation factor 88, MyD88 [16], C-type lectin [17] and Cactus Gene [18]) of the pacific white shrimp, *Litopenaeus vannamei* showed different expression patterns when challenged with WSSV and poly I: C. These comparison studies may help to better understand the role of intestinal immune system in response to various potential pathogens in crustaceans.

Herein, a variety of markers potentially useful for genomic population studies including SSRs located within coding regions and SNPs detected amongst deep coverage sequence region reads were also reported. Similar studies have been reported in several crustaceans. In shrimp *L*. *vannamei*, the prospected SNPs spread out among 25,071 unigenes and allocated to 254 pathways at the KEGG [19]. In the black tiger shrimp *Penaeus monodon*, a high density linkage map was built and believed to be causal or closely related to other mutations that affect the resistance to diseases [20]. In the freshwater crayfish *Procambarus clarkii*, SSRs and SNP markers were generated from hepatopancreas, muscle, ovary, and testis, which may represent a resource for trait mapping [21]. In this prawn *M*. *rosenbergii*, a number of potential SSR and SNP markers has been also isolated from the tissues of androgenic gland, eyestalk, gill, heart, ovary, testis, hepatopancreas and muscle in healthy prawn [5]. However, relatively few data were available about the SNP or SSRs from intestine tissue upon WSSV or poly (I:C) challenges. The huge number of potential SSR and SNP markers identified in this study may shed the lights on developing disease resistance breeding projects of *Macrobrachium* species.

Regarding Gene Ontology (GO) categories of the combined unigenes and DEGs, results here were similar with the studies in penaeid. Considering the biological processes, per instance, the most frequent were cellular process and metabolic process. In what regards cellular components, genes are mostly expressed at the cell and some unspecific organelles. Finally, concerning the molecular function, the most common ones were catabolic activity and binding [19, 20]. Compared with the transcriptome profiling of the *M*. *rosenbergii* lymphoid organ, the intestine had similar top GO terms but significantly different KEGG pathway enrichments when challenged with WSSV [3], which may indicate the different roles of intestine and lymphoid organ in prawn innate immune systems.

In *M*. *rosenbergii* hepatopancreas, after WSSV infection, 8443 unigenes significantly up-regulated and 5973 unigenes significantly down-regulated [4]. In lymphoid organ, 4055 were up-regulated, and 896 were down-regulated [3]. Similarly, here in the intestine, after WSSV or poly (I:C) treatments, the up-regulated DEGs were also much greater than the down-regulated genes (Fig 3A). It could be hypothesized that the virus infection in the prawn was associated with the accumulation of novel transcripts, and these DEGs may play an important role in the signaling transduction of elimination of external stimulus.

Ingestion of WSSV-infected prawn has been accepted as the major route of natural infection due to the cannibalistic nature of many crustaceans [22]. Epithelium of the intestinal midgut is generally lined with the peritrophic membrane (PM), which is a noncellular structure surrounding the food bolus. Proteoglycans were considered to be the main component of PM. Therefore, WSSV must cross the PM in the midgut to traverse the basal membranes and reach the host cells [23]. In this study, in the intestines of the prawn *M*. *rosenbergii*, genes of the proteoglycans related pathway expressed significantly differently after WSSV or poly I:C challenge. We speculate that the interaction between WSSV and proteoglycans may be important for WSSV infection in *M*. *rosenbergii*. Considering that proteoglycans have been accepted as a major role in preventing or controlling infectious microbes [24], results here may also provide some data for developing the virus prevention strategies.

Another interesting phenomenon was that genes in the spliceosome pathway also expressed differently following WSSV and poly (I:C) challenge. Many human diseases were associated with the aberrant change in spliceosome components, which may cause splicing defects or alterations [25, 26]. In penaeid, spliceosome was considered to be one of the most commonly described pathways involved in the taura syndrome virus (TSV) and WSSV infection [27]. In freshwater crayfish *P*. *clarkii*, spliceosome was also on the list of potential antiviral signaling pathways [28]. Similarly, in RNA-seq analysis of *M*. *rosenbergii* hepatopancreas in response to *Vibrio parahaemolyticus* and WSSV infection, the majority of the unigenes fell into the categories of spliceosome pathway [5]. Additionally, spliceosomes and the RNA transport pathway supposedly act in the formation of new transcripts, providing genetic variants that may contribute to resistance [29]. However, there is still much work to do to study the precise functions of genes in the spliceosome pathway.

The Warburg effect (or aerobic glycolysis) was a metabolic shift that first found in cancer cells [30], but recently it was discovered both in vertebrate and invertebrate cells infected by viruses [31]. The Warburg effect facilitated the production of more energy and building blocks to meet the enormous biosynthetic requirements of cancerous and virus-infected cells. Recent research suggested that WSSV triggers Warburg effect via the PI3K-Akt-mTOR pathway in shrimp *L*. *vannamei* [31]. Herein, the comparative transcriptome results of WSSV and poly (I:C) treatments in *M*. *rosenbergii* revealed that PI3K-Akt-mTOR exhibited significantly different expression (Fig 4), confirming that this pathway was of central importance in triggering the WSSV-induced Warburg effect and essential for successful viral replication.

## Conclusion

The interaction between the intestine immune system and WSSV or virus mimic in freshwater prawn *M*. *rosenbergii* was investigated. Deep analysis of the transcriptome comparative data including DEG functional annotation, orthologous protein clustering, and annotation of signaling pathways determined the anti-viral intestine immune response in *M*. *rosenbergii*. More functional analysis will be needed to fully elucidate the specific roles of DEGs and the underlying immune defense mechanisms of *M*. *rosenbergii*.

## Supporting information

S1 Fig Functional distribution of KEGG annotation of the whole unigenes identified from intestines of WG, PG and NG prawns *Macrobrachium rosenbergii*. X axis represents the number of Unigenes. Y axis represents the KEGG functional category.
(TIF)

S2 Fig Visualization of differentially expressed genes (DEGs) between infection and mock groups. A, MA plot of DEGs between NG (normal group) and WG (48 h post WSSV infection). B, MA plot of DEGs between NG (normal group) and PG (48 h post Poly I:C challenge). X axis represents log2 transformed mean expression level; Y axis represents value log2 transformed fold change. C, Heatmap of hierarchical clustering of DEGs. X axis represents each comparing samples. Y axis represents DEGs. Coloring indicate fold change (high: red, low: blue).
(TIF)
