# Supplementary figures and images for: Transcriptome analysis of Macrobrachium rosenbergii intestines under the white spot syndrome virus and poly (I:C) challenges
Source: PLoS One. 2018 Sep 28;13(9):e0204626. doi: 10.1371/journal.pone.0204626 (PMC6161888; doi:10.1371/journal.pone.0204626)

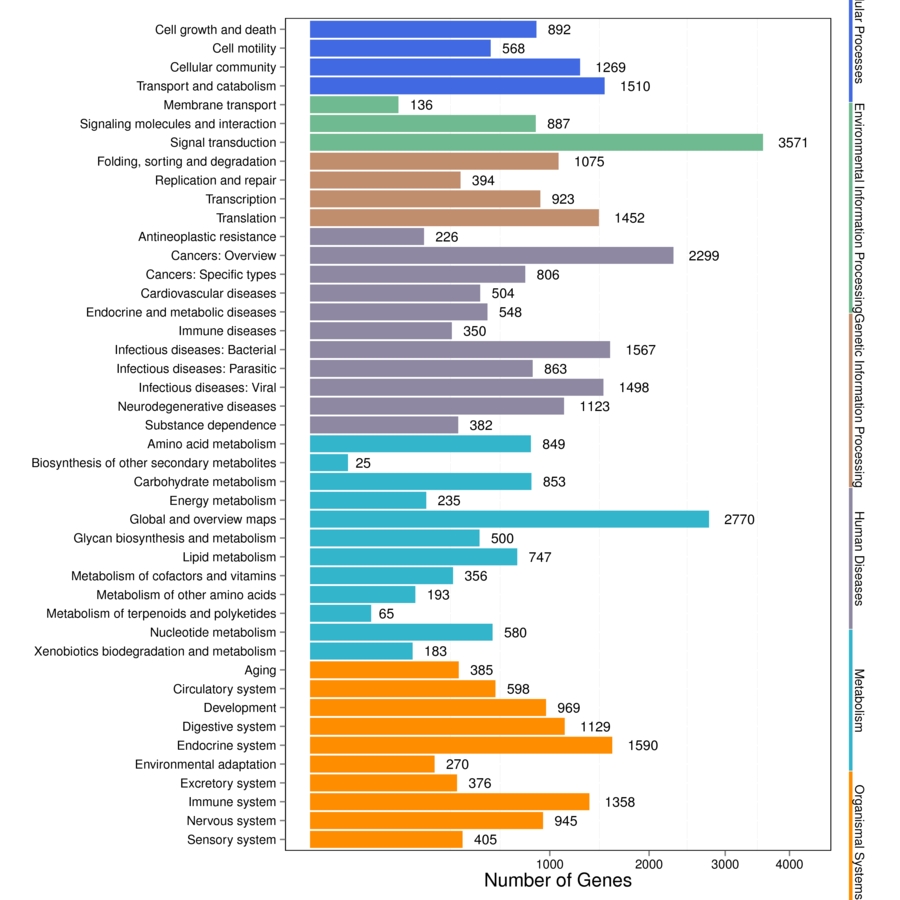

Supplement: S1 Fig — X axis represents the number of Unigenes. Y axis represents the KEGG functional category. (TIF) [file pone.0204626.s001.tif]

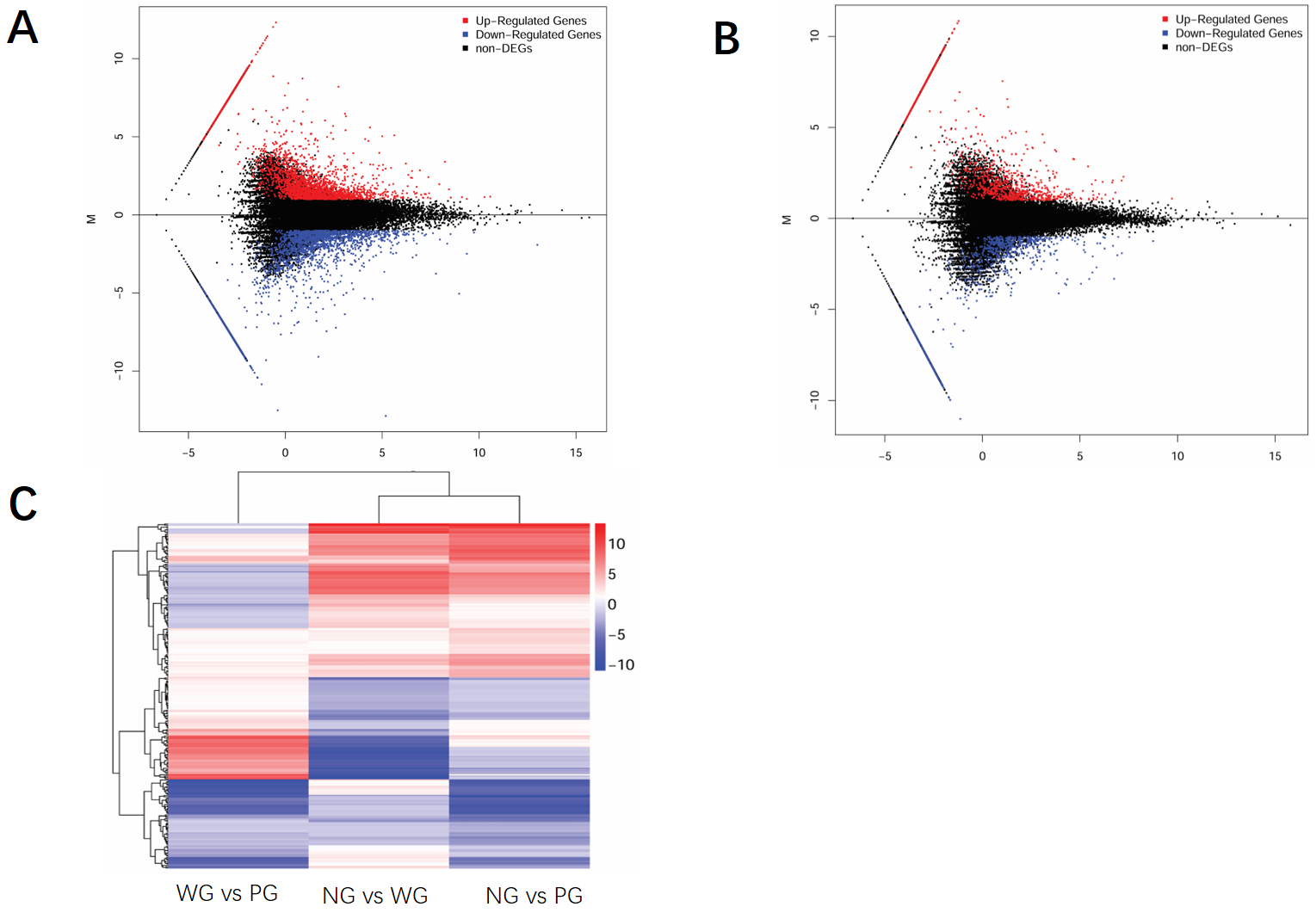

Supplement: S2 Fig — A, MA plot of DEGs between NG (normal group) and WG (48 h post WSSV infection). B, MA plot of DEGs between NG (normal group) and PG (48 h post Poly I:C challenge). X axis represents log2 transformed mean expression level; Y axis represents value log2 transformed fold change. C, Heatmap of hierarchical clustering of DEGs. X axis represents each comparing samples. Y axis represents DEGs. Coloring indicate fold change (high: red, low: blue). (TIF) [file pone.0204626.s002.tif]
